# Cancer-Associated Fibroblasts Connect Metastasis-Promoting Communication in Colorectal Cancer

**DOI:** 10.3389/fonc.2015.00063

**Published:** 2015-03-23

**Authors:** Joke Tommelein, Laurine Verset, Tom Boterberg, Pieter Demetter, Marc Bracke, Olivier De Wever

**Affiliations:** ^1^Laboratory of Experimental Cancer Research, Department of Radiation Oncology and Experimental Cancer Research, Ghent University, Ghent, Belgium; ^2^Department of Pathology, Erasme University Hospital, Université Libre de Bruxelles, Brussels, Belgium

**Keywords:** cancer-associated fibroblasts, pericryptal fibroblasts, colorectal cancer, metastasis, immune cells

## Abstract

Colorectal cancer (CRC) progression and eventually metastasis is directed in many aspects by a circuitous ecosystem consisting of an extracellular matrix scaffold populated by cancer-associated fibroblasts (CAFs), endothelial cells, and diverse immune cells. CAFs are recruited from local tissue-resident fibroblasts or pericryptal fibroblasts and distant fibroblast precursors. CAFs are highly abundant in CRC. In this review, we apply the metastasis-promoting communication of colorectal CAFs to 10 cancer hallmarks described by Hanahan and Weinberg. CAFs influence innate and adaptive tumor immune responses. Using datasets from previously published work, we re-explore the potential messages implicated in this process. Fibroblasts present in metastasis (metastasis-associated fibroblasts) from CRC may have other characteristics and functional roles than CAFs in the primary tumor. Since CAFs connect metastasis-promoting communication, CAF markers are potential prognostic biomarkers. CAFs and their products are possible targets for novel therapeutic strategies.

## Introduction

Colorectal cancer (CRC) is the third most common cancer and a major cause of mortality in the western society. The 5-year survival rate is approximately 66.1% for all stages (1). However, prognosis is strongly related to stage at diagnosis. The 5-year relative survival rates are 90% for localized disease compared with 13% for patients with distant metastases at diagnosis (2). The major cause of death in this malignancy is development of metastases in liver and lung. The majority of patients with metastatic CRC remain incurable and currently have a median survival of 2 years (3).

In the past decades, treatment and molecular characteristics relating to prognosis of CRC were mainly focused on the malignant cancer cells. However, recent studies revealed that CRC progression is directed in many aspects by a circuitous ecosystem consisting of an extracellular matrix (ECM) scaffold populated by cancer-associated fibroblasts (CAFs), vascular space-related cells (endothelial cells, pericytes, and smooth-muscle cells), and diverse innate and adaptive immune response cells (lymphocytes, macrophages, and mast cells) (4). In this review, we discuss the role of CAFs in CRC and their tumor-promoting characteristics on the basis of the 10 hallmarks of cancer of Hanahan and Weinberg (5). To better understand the role of CAFs in CRC, we first explore the role of resident fibroblasts under healthy physiological circumstances in the colon. Despite the evidence of the role of CAFs in tumor progression, we explore the possibility that CAFs may have tumor-inhibiting effects as well. Finally, we discuss the role of fibroblasts in metastases of CRC.

## Fibroblasts in the Normal Colon

In the normal colon, fibroblasts are spread throughout the lamina propria adjacent to the colon mucosal epithelium. These fibroblasts are in a resting state and are α-smooth-muscle actin (α-SMA) negative (6). Pericryptal fibroblasts (PCFs) express α-SMA, smooth-muscle myosin, vimentin, and are desmin-negative, defining them as myofibroblasts (7). These are mesenchymal cells that express features of fibroblast and smooth-muscle differentiation (8). PCFs are located subjacent to the crypt epithelial cells and follow the contours of the wall of the crypt (9). This enables PCFs to establish and maintain instructive communications with colonic stem cells and their descendants (10). PCFs maintain a parallel relationship in replication, differentiation, and migration with the overlying epithelium (9). The interaction between these cells helps to maintain tissue integrity (11). PCFs deposit type IV collagen necessary for the production of the pericryptal basement membrane (6). PCFs are important for epithelial differentiation during fetal and adult life and for the absorptive function of the crypts, interacting with epithelial cells through a paracrine mechanism by transforming growth factor-β (TGF-β), interleukin (IL)-6, and leukemia inhibitory factor (LIF) (12). Furthermore, PCFs are implicated in the regulation of mucosal inflammation. Through their secretion of cytokines, chemokines, eicosanoids, and ECM components, stromal cells affect the recruitment, retention, and activation of immune cells. PCFs exhibit class II major histocompatibility complex expression and are strategically located at the interface between the epithelium and lymphocytes of the lamina propria. Therefore, PCFs may be among the first cells to interact with antigens that cross the epithelial barrier and present them to lamina propria CD4^+^ T-cells, which initiate and maintain adaptive immunity (8). In the colonic mucosa, there are three major populations of mesenchymal cells: PCFs, lamina propria fibroblasts, and smooth-muscle cells of the muscularis mucosae. These cell populations are interconnected, forming a complex three-dimensional scaffold. PCFs connect to the cells of the muscularis mucosae at the base of the crypts, whereas they also form a syncytium with the lamina propria fibroblasts, which is in turn linked with the pericytes of the mucosal capillaries (7).

## Fibroblasts in CRC

### Colon CAFs

Fibroblasts of the tumor stroma are called activated fibroblasts, myofibroblasts, tumor-associated fibroblasts, or CAFs. They are the main cellular constituents of stroma associated with primary and metastatic CRC (4, 13). Commonly used markers to identify CAFs are α-SMA, fibroblast activation protein-α (FAP-α), fibroblast-specific protein-1 (FSP-1/S100A4), or platelet-derived growth factor receptor-β (PDGFR-β) (14). Individually, these markers could identify specific subpopulations of fibroblasts, thus it would be more correct to use a combination of markers to select the largest possible population of CAFs (15). α-SMA has been demonstrated not to label CAFs exclusively, but also smooth-muscle cells in the muscularis mucosae and muscularis propria (16, 17). FAP appears to be expressed on pericytes and CAFs (17). Quiescent resident fibroblasts express vimentin, instead of α-SMA, as intermediate filament proteins (18). CAFs are negative for smooth-muscle markers smoothelin and desmin and positive for the marker CD90, which is not expressed in T-lymphocytes in human (8). CAF cultures maintain the phenotypic characteristics of CAFs even in absence of interaction with cancer cells (4).

The number of myofibroblasts is strongly increased in CRC compared to normal mucosa. It was demonstrated that lamina propria fibroblasts change from α-SMA^−^ to α-SMA^+^ in colorectal polyps, suggesting that interstitial lamina propria fibroblasts show myofibroblast differentiation (7). Accordingly, genes up-regulated in the tumor stroma versus normal stroma encompassed CAF markers, such as PDGFR-β or FAP (14).

The origin of CAFs is diverse and still under investigation. The majority of CAFs are generated by differentiation of resident fibroblasts. It is suggested that cancer cells recruit fibroblasts and secrete factors that promote their myofibroblast differentiation, as part of prominent fibrosis known as tumor desmoplasia (19). Desmoplasia consists of CAFs as key cellular component and is a pathologic feature of human solid cancers and their metastases (13, 20). Several cytokines including TGF-β, platelet-derived growth factor (PDGF), IL-4, IL-6, insulin-like growth factor (IGF)-II, and prostaglandin E (PGE) have been reported to induce CAF differentiation (3, 21–23). A second source of CAFs are mesenchymal stem cells (MSCs) residing in the bone marrow. MSCs are multipotent non-hematopoietic cells that can differentiate into different types of mesenchymal cells (21). MSCs were shown to be attracted to the tumor microenvironment, where they proliferate and become CAFs (17). Differential secretome profiling between CAFs from CRC compared to bone marrow-derived MSCs (BM-MSCs) demonstrated that 52.5% of proteins were detected in both samples. It has been revealed that treatment of BM-MSCs with recombinant TGF-β1 induced a CAF-phenotype with increased α-SMA expression, stress fiber organization, and loss of multipotency (24). Recombinant TGF-β1 induced secretion of 84 proteins in MSCs, of which 16 proteins were also present in the CAF secretome. This indicates that additional factors probably act in cooperation with TGF-β1 to induce a fully differentiated MSC phenotype or suggests several precursors of CAFs (24). Indeed, experimental data propose a number of other cell types that may be a source of CAFs, including adipocytes, stellate cells, pericytes, circulating mesenchymal or hematopoietic stem cells, and CD34-positive fibrocytes (3, 23). CAF differentiation is stimulated by reactive oxygen species (ROS), acting through both TGF-β1-dependent and -independent mechanisms. Less is known about factors inhibiting CAF differentiation. The inflammatory cytokines interferon (IFN)-γ and tumor necrosis factor (TNF)-α inhibit differentiation (3). One of the CAF recruitment mechanisms by cancer cells is increased expression of tissue inhibitors of metalloproteinases (TIMP)-1. Stroma of human prostate and colon cancer expresses higher levels of TIMP-1 compared to its phenotypically normal counterpart. TIMP-1 leads to increased CAF proliferation and migration through binding of TIMP-1 to its receptor CD63, which is expressed on CAFs (25).

### Tumor-promoting characteristics of CAFs

**Figure 1:**
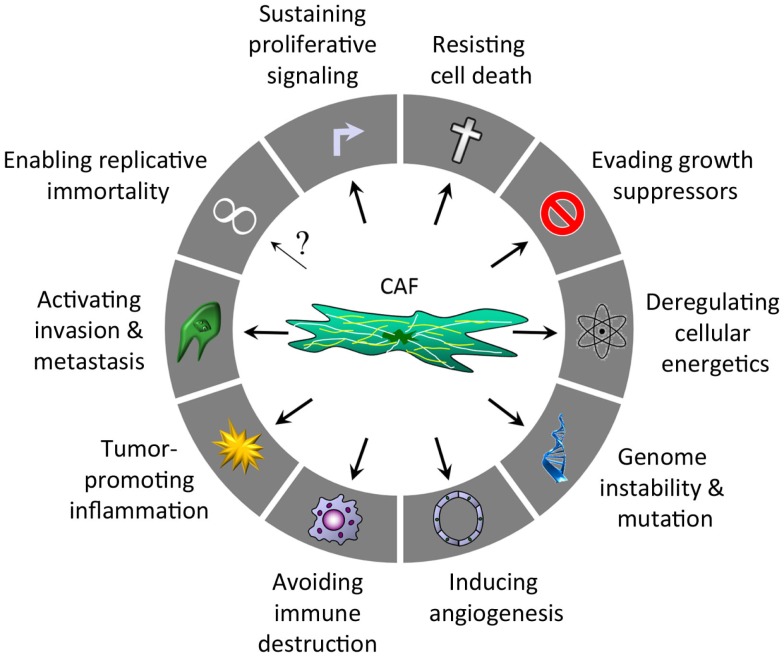
**Hallmarks of cancer regulated by CAFs after Hanahan and Weinberg (5)**.

In 2000, Hanahan and Weinberg identified six hallmarks of cancer, primarily focusing on the phenotypic changes required for normal tissue to become cancerous. A decade later new insights led to two emerging hallmarks (reprograming of energy metabolism and evading immune destruction) and two new enabling characteristics (tumor-promoting inflammation and genome instability and mutation) (5). Many of these hallmarks are interconnected and the role of CAFs is described below (Figure 1).

#### Sustaining proliferative signaling, resisting cell death, and evading growth suppressors

Multiple CAF-derived factors sustain proliferative signaling in CRC cells and support the cancer cells to resist cell death and evade growth suppressors. Colon CAFs secrete epidermal growth factor (EGF), hepatocyte growth factor (HGF), IGF1/2, PGE-2, PDGF, fibroblast growth factor (FGF)-1, and vascular endothelial growth factor (VEGF) (20, 21, 24). These growth factors act through activation of the mitogen-activated protein kinase (MAPK) and phosphatidylinositol 3-kinase (PI3K)/AKT pathways, which mediate cell proliferation and cell survival (anti-apoptotic signaling), protein synthesis, cytoskeletal rearrangements, and invasion (26). For example, it was demonstrated that HGF and neuregulin-1 in supernatants of CAFs were able to phosphorylate the receptor tyrosine kinase c-Met and human epidermal growth factor receptor (HER)-2/HER-3 in CRC cells thereby activating both the MAPK and PI3K/AKT pathway. Consequently, supernatants of CAFs induced an increase in CRC cell counts and tumorigenicity (27, 28).

CAF-derived TGF-β and connective tissue growth factor (CTGF) leads to proliferation through the Smad2/Smad4 pathway (20, 21, 24, 29). The interaction of cancer cells with CAF-derived factors initiates TGF-β signaling and growth in CRC cells with a functional TGF-β receptor complex, possibly through activation of latent TGF-β1 (22). The putative TGF-β-target IGFBP-7 was up-regulated in CAFs compared to normal resting colonic fibroblasts. Although IGFBP-7 is a known tumor suppressor, CAFs expressing IGFBP-7 stimulate colony formation of co-cultured CRC cells, suggesting that IGFBP-7 indirectly promotes tumor progression through manipulation of CAFs (14).

The ECM protein periostin is secreted by PCFs and CAFs in the colon. Both colony size and number of HCT116 cells were significantly larger when cultured with periostin-producing fibroblasts than with non-producing ones in a three-dimensional co-culture system (12). Epiregulin expression is up-regulated in patients with colitis-associated cancer. CAFs were identified as major source. CAF-derived epiregulin requires ERK activation to induce proliferation of CRC cells and tumor development *in vivo* (30). CD133^+^ CRC cells exhibit enhanced tumorigenicity over CD133^−^ cells. Up-regulated genes in CD133^+^ cells included chemokine receptor CXCR4, integrin β8, and fibroblast growth factor receptor (FGFR)-2. The adjacent CAFs overexpress the gene transcripts for the activating ligands: stromal-derived factor-1 [SDF-1; also known as C–X–C motif ligand 12 (CXCL-12)], macrophage migration inhibitory factor (MIF), vitronectin, and FGF family members. In matrigel three-dimensional culture, the CD133^+^/CXCR4^+^ group treated with recombinant SDF-1 led to more and larger colonies compared with vehicle. CD133^+^ cells are spatially located next to a relative abundance of CAFs in CRC. Thus, the enhanced tumorigenic potential of CD133^+^ cells possibly origins in their increased ability to interact with their neighboring CAFs (31).

Two prototypical tumor/growth suppressors are the retinoblastoma (RB)-associated and TP53 proteins. The p53 gene (TP53) encodes a transcription factor that acts as a tumor suppressor and can be activated in response to oncogenic stress signals. Once activated, p53 induces apoptosis or replicative senescence in order to prevent the proliferation of potentially malignant cells. p53 induction alters CAFs’ protein secretion, leading to a less supportive microenvironment. For instance, p53 can repress the expression of the chemokine SDF-1 in fibroblasts. In agreement with a tumor suppressor role for p53 in CAFs, lung cancer cells suppress p53 induction in CAFs, by a mechanism that is independent of direct cell–cell contacts. Human CAFs are more susceptible to this inhibitory mechanism than their normal counterparts (32). The RB protein is hypo-phosphorylated in quiescent or differentiated cells, it interacts with E2F family transcription factors, repressing the transcription of genes essential for cell cycle progression. In isolated breast CAFs, RB protein is inactivated by phosphorylation, consistent with cell cycle progression (33). In accordance, oro-pharyngeal cancer specimens were analyzed by immunohistochemical staining for phosphorylated RB protein, demonstrating that RB and RB-dependent pathways were generally inactivated in CAFs. Depletion of RB in CAFs enhances the invasive potential of cancer cells through increased production of CAF-derived keratinocyte growth factor (KGF or FGF7), that regulates invasion via an AKT–Ets2–matrix metalloproteinase (MMP)-1-dependent pathway (34). Further studies are necessary to conclude about the role of p53 or RB in colon CAFs.

#### Deregulating cellular energetics and genome instability and mutation

The role of CAFs in deregulating cellular energetics has been reviewed by Martinez-Outschoorn et al. (35). Metabolic connection occurs between catabolic fibroblasts and anabolic cancer cells. In brief, altered cell signaling pathways in cancer cells drive the generation of ROS and autophagy in adjacent CAFs. In turn, catabolic CAFs fuel tumor growth through energy and biomass transfer to anabolic cancer cells. CAF catabolism is associated with poor clinical outcome (35).

Immunohistochemical evaluation of colorectal adenocarcinomas revealed a reduced pyruvate dehydrogenase (PDH) expression and a high lactate dehydrogenase-5 (LDH-5) isoenzyme and PDH-kinase1 (PDK1) reactivity in CRC cells (36). PDK1 is an enzyme inhibiting PDH activity. Thus, cancer cells are directed to anaerobic glycolysis for energy, which is mediated by pyruvate transformation to lactic acid via the catalytic activity of LDH-5. Cellular metabolism shifts to anaerobic pathways for ATP production when there is only suboptimal oxygen availability. However, cancer cells show an intrinsic trend to switch to glycolysis even in the presence of high oxygen, the so-called Warburg effect. In contrast, CAFs maintain a rather strong PDH expression whereas reduced LDH-5 and PDK1 activity is noticed. This indicates metabolism proceeding to pyruvate followed by oxidative phosphorylation in the mitochondria. CAFs demonstrate a widespread localization of the LDH-1 isoenzyme, which has reduced ability to convert pyruvate to lactate compared with LDH-5, thereby promoting oxidation of lactate back to pyruvate. The stroma forms a major buffer of acidity through the absorption of lactic acid released by CRC cells and recycling it back to pyruvate, sustaining cancer cell survival (36). CRC cells close to CAFs have a high proliferative index, high HIF-1a and LDH-5 reactivity, and a tendency to extramural extension. Lactate increases intracellular oxidants, promoting proliferation of cultured fibroblasts (37). CAFs express proteins involved in lactate absorption, lactate oxidation, and reduced glucose absorption. Therefore, CAFs use lactate for energy and save glucose for cancer cells, which are dependent upon anaerobic metabolism with a high ability for glucose absorption. In conclusion, the complementary metabolic function of cancer cells and CAFs shows synergistic support to each other (36).

Metabolic coupling and mutagenic co-evolution of epithelial cancer cells and CAFs during tumor formation was demonstrated in a co-culture of normal human fibroblasts with MCF-7 breast cancer cells. Breast cancer cells induce ROS production and a loss of caveolin-1 in adjacent fibroblasts, leading to a CAF-phenotype. The loss of caveolin-1 in fibroblasts triggers nitric oxide (NO) production, mitochondrial dysfunction, and oxidative stress via ROS production. Oxidative stress promotes DNA damage and genetic instability in cancer cells, potentially increasing their aggressive behavior (mutagenic evolution) by a bystander effect (38). Similarly, CRC cells can acquire genetic alterations by an oxidative stress induced bystander effect. It was demonstrated that infection by *Enterococcus faecalis*, a constituent of the intestinal microbiota known to generate ROS, induced genetic instability and aneuploidy in colonic epithelial cells (39). On the other hand, oxidative stress due to loss of caveolin-1 triggers aerobic glycolysis in breast CAFs, which is in contrast to the results obtained in CRC. According to “The Reverse Warburg Effect,” cancer cells and stromal fibroblasts dynamically co-evolve and become metabolically coupled during tumor formation, inducing aerobic glycolysis in CAFs by cancer cells. As a consequence, these CAFs secrete high levels of energy-rich metabolites (such as lactate and pyruvate) that are directly absorbed by tumor cells and used for efficient ATP production via oxidative phosphorylation (38). However, these data remain limited to CAFs activated by deletion/down-regulation of caveolin-1 and are not reported for CRC.

#### Inducing angiogenesis

Colon CAFs produce significant amounts of IL-6 and CRC cells further enhance IL-6 production by CAFs. IL-6 is a multifunctional cytokine that plays a central role in the regulation of inflammatory and immune responses, but it is also characterized as an angiogenic cytokine. RT-PCR and ELISA demonstrated that IL-6 up-regulated the expression of VEGFA mRNA and VEGFA protein from both normal colon fibroblasts and CAFs. IL-6 was suggested to stimulate VEGF secretion by the mediation of PGE-2 from CAFs. Secreted VEGF from fibroblasts targets endothelial cells and is known as one of the most important angiogenic factors. Co-cultures of colon fibroblasts and human umbilical vein endothelial cells were used as an angiogenesis model. Immunostaining with anti-CD63 antibody showed increased vascular area and vascular network formation after treatment with recombinant IL-6 (40). Co-cultures of CRC cells with CAFs revealed a synergistic increase of VEGF production compared with monocultures. The expression of VEGF mRNA in CAFs was significantly increased by co-culture with CRC cells, suggesting that the large amounts of VEGF detected in the supernatants of co-cultures were mainly derived from CAFs (41). Follistatin-like protein-1 (FSTL-1) expression was observed in the human CRC stromal compartment. FSTL-1 is a TGF-β-inducible gene, secreted protein and acidic and rich in cysteine (SPARC)-related, that enhances inflammatory cytokine/chemokine expression and also seems to be implicated in angiogenesis and revascularization (29).

To examine which other proteins secreted by CAFs are important for angiogenesis, we analyzed data of De Boeck et al. Proteins secreted by CAFs, MSCs, or recombinant TGF-β1-treated MSCs were reported (24). We specifically studied the proteins uniquely secreted by CAFs and by the combination of CAFs and recombinant TGF-β1-treated MSCs, which are not secreted by non-treated MSCs. Re-analysis of these proteins through the functional enrichment analysis tool (FunRich – http://www.funrich.org/) revealed that 19.44% is implicated in angiogenesis (Table 1).

**Table 1:** **Angiogenesis-related proteins secreted uniquely by CAFs from CRC and by the combination of CAFs and recombinant TGF-β1-treated MSCs according to their category**.

Cytokine/chemokine activity	Growth factor	Others
GDF-15	EGF	PPP2CA
TGFB-2	HGF	PPP2R1A
CCL-5	FGF-2	CLTB
CXCL-12	IGF-1	LRP-1
CCL-11		MMP-3
CSF-1		PGM-1
CSF-2		CD44
IFNG		MFGE-8
		PA2G4
		UBE2D3
		NRP-2
		IGFBP-1
		AGT
		MAPK
		RUVBL-1
		HSPD-1

CAF-derived chemokine SDF-1 (CXCL-12) is reported to recruit endothelial progenitor cells into breast carcinomas to induce angiogenesis. Breast cancer cells mixed with CAFs in a xenograft model led to highly vascularized tumors (42). IFN-γ has immunomodulatory and other anti-tumor activities. Selective targeting of IFN-γ to CAFs and pericytes through a PDGFβR-binding carrier, because these cells express high levels of PDGFβR, reduces tumor growth by inhibition of angiogenesis. In mice subcutaneously injected with B16-F10 melanoma cells, α-SMA^+^ cells (fibroblasts and pericytes) were reduced in targeted IFN-γ-treated tumors. CD31 staining of the lumen area of tumor blood vessels demonstrated a significant reduction in angiogenesis (43). IL-6 and FSTL-1 are not present in the FunRich results, because these proteins are also secreted by non-treated MSCs, but their secretion is increased in CRC (24).

TIMP-1 is a prognostic marker in plasma from CRC patients. Higher levels of TIMP-1 were present in patients with metastases. In liver metastases, TIMP-1 was expressed by α-SMA^+^-activated hepatic stellate cells (HSCs) situated adjacent to CD34-positive endothelial cells, suggesting a function in tumor-induced angiogenesis (44). In an orthotopic nude mice model, KM12SM human CRC cells mixed with MSCs were injected into the cecal wall. CD31 staining of the primary tumor demonstrated that the microvessel area was significantly greater in the KM12SM + MSCs group than in the KM12SM-alone group. When mice were treated with imatinib, which is a tyrosine kinase inhibitor of PDGFR, there was no difference anymore between the two groups (45, 46).

#### Avoiding immune destruction

The immune system plays a considerable role in tumor development. Tumor progression and spread is sustained by the immunoediting hypothesis in which the tumor environment is selected for escape from the immune system (19). Major escape mechanisms to anti-tumor immune response are induction of regulatory T (T_reg_) cells or immunosuppressive molecules such as programed cell death protein-1 or cytotoxic T-lymphocyte (CTL)-associated protein-4 on CTLs and down-regulation of antigen-presenting molecules (47). T_reg_ cells are CD4^+^, CD25^+^, Foxp3^+^ immunosuppressive lymphocytes that suppress autoreactive T-cells to maintain immunological self-tolerance and inhibit autoimmunity (48). T_reg_ cells secrete immunosuppressive cytokines, as IL-10 and TGF-β, and metabolites such as adenosine (47).

Effector cells responsible for the immune response in the tumor stroma include on the one hand tumor-promoting regulatory dendritic cells (DCs), M2 macrophages, N2 neutrophils, myeloid DCs, and T_reg_ cells; and on the other hand, tumor-inhibiting classic DCs, M1 macrophages, N1 neutrophils, CTLs, natural killer (NK) cells, and natural killer T (NKT) cells (Table 2) (19, 47). Tumor and CAF secretion of chemokines can recruit immunosuppressive myeloid-derived suppressor cell population (MDSC) to the tumor. MDSC comprise neutrophils, immature DCs, monocytes, and early myeloid progenitors. They not only suppress adaptive immunity, but also promote angiogenesis through the secretion of VEGFA, bFGF, and TGF-β. MDSCs also inhibit NK cell function and enlarge the T_reg_ cell population. In addition, MDSCs can directly inhibit effector T (T_eff_, CD4^+^, Foxp3^−^) cell growth, activation, and migration by altering the environment (Table 2) (49). Cells such as MDSC, T_reg_ cells, and M2 tumor-associated macrophages (TAMs) are recruited or differentiated in the tumor microenvironment. These cells further enhance the helper T-cell-2 polarization often synonym of CTL suppression and cancer cell dissemination (17).

**Table 2:** **Tumor-promoting versus tumor-inhibiting immune cells**.

Tumor-promoting	Tumor-inhibiting
CD4^+^, CD25^+^, Foxp3^+^ T_reg_ cells	CD8^+^ CTLs
MDSC	NK cells
Regulatory DCs	NKT cells
Myeloid DCs	Classic DCs
M2 macrophages/monocytes	M1 macrophages
N2 neutrophils	N1 neutrophils

The regulation of the anti-tumor immune response by CAFs has recently been reviewed by Harper and Sainson (17). To study the role of CAFs from CRC in immune response, we used previously published data from our group to re-analyze proteins secreted uniquely by CAFs and by the combination of CAFs and recombinant TGF-β1-treated MSCs, which are not secreted by non-treated MSCs (24). To reveal if some of these proteins were related to immunity, we used FunRich (http://www.funrich.org/) which gives the opportunity to cluster the secreted proteins by biological processes or pathways. Approximately 1/5 of all secreted proteins are implicated in immune system, immune response, or immune-related pathways, such as IFN-γ signaling and chemokine response (Table 3).

**Table 3:** **Immune-related proteins secreted uniquely by CAFs from CRC and by the combination of CAFs and recombinant TGF-β1-treated MSCs according to their category**.

Chemokine response	Interferon signaling	Mixed immune system
CCL-5	CD44	CSF-2
CXCL-12	PPP2CA	MAPK-1
CCL-11	PPP2R1A	UBE2D3
CCL-7	GBP-1	CALR
CXCL-16	GBP-2	CD14
	IFNG	C3
	ISG-15	PSMD-7
		PSMD-3
		PSMD-8
		CXCL-6
		CD59
		CSF-1
		CYTL-1
		MR-1
		HSPD-1

**Figure 2:**
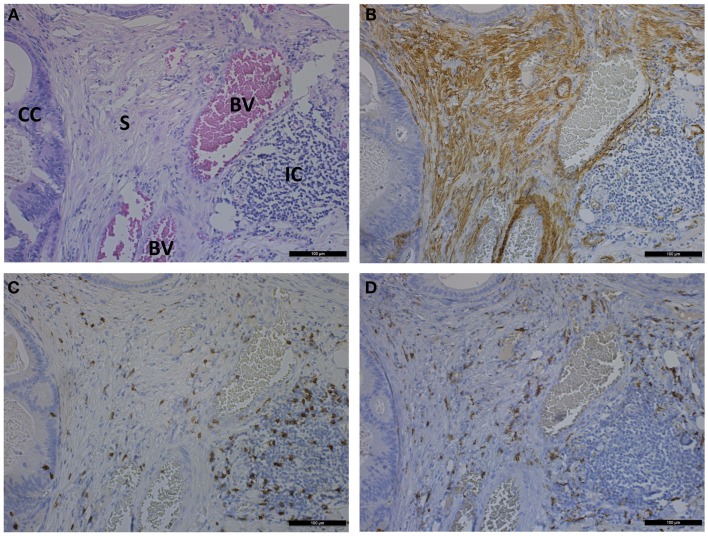
**Photomicrographs showing hematoxylin and eosin staining (A) and immunohistochemical staining of α-SMA (B), CD8 (C), and CD163 (D) on consecutive sections of a primary colon tumor**. Scale bar is 100 μm. CC, cancer cells; S, stroma; BV, blood vessel; IC, immune cells.

In CRC development, the immune system plays a complex role. Although chronic inflammation, including inflammatory bowel disease, can be a precursor to CRC development, a distinct anti-tumor CD3-positive T-cell response is the best predictor of long-term survival for CRC patients. In CRC, a pronounced desmoplastic reaction is associated with a reduced immune response. Therefore, CAFs may play a role in tumor immune evasion (3). In corresponding tumor tissue sections from the same patients, the amount of intercellular adhesion molecule (ICAM)-1-positive CAFs was significantly higher than that in the corresponding normal colon mucosa. These results indicate that CAFs from CRC tissue exhibit an increased affinity for monocytic cells. This increased intercellular interaction may contribute to extended residence times of monocytes in CRC tissue (50). Co-expression of CAF and M2 macrophage markers is associated with the clinical outcome of CRC patients. TAMs can be present in the tumor in two phenotypically different populations. M1 macrophages will develop an anti-tumor response by producing inflammatory molecules (IL-6, IL-12, IL-23, and TNF-α) and ROS, NO, and TNF. M2 macrophages secrete immunosuppressive cytokines (IL-10 and TGF-β) and promote tumor progression by stimulating angiogenesis and degradation of the ECM (15, 47). M2 macrophages are CD163^+^ and correlate with CAF markers. Multivariate analysis demonstrates that the combination of both variables is an independent prognostic marker for poor disease-free survival and overall survival and which is statistically stronger than either of both alone (15). In agreement, CD163 immunohistochemical staining on patient sections of primary colon tumors revealed a high presence of TAM in zones with high presence of α-SMA-positive CAFs (Figure 2). This suggests a correlated expression pattern of both M2 macrophages and CAFs. CD8 immunohistochemical staining illustrated that T-lymphocytes are widespread and infiltrate the cancer cells (Figure 2).

Heterogeneity of the tumor immune context is influenced by various factors, including those secreted by CAFs and the cancer cells themselves. For instance, microsatellite unstable CRCs have a more intensive CTL infiltration and better prognosis than microsatellite stable CRCs (47). On the other hand, IL-11, a TGF-β target gene in CAFs, contributes to a survival advantage to metastatic cells. IL-11 stimulates platelet production. Platelets protect circulating cancer cells from immune attacks, as well as support them during extravasation. In addition, platelets are a rich source of TGF-β (51). IL-6 and PGE-2 derived from colon CAFs were identified as potential immune-suppressive molecules (14, 21, 29). In co-culture experiments, CAFs from CRC intensely suppressed the expression of the NK receptors, perforin and granzyme B, but they also inhibited the secretion of the cytokines TNF-α and IFN-γ by NK cells. In the presence of NK cells, CAFs produced pro-inflammatory lipid mediator PGE-2 at higher levels than fibroblasts from healthy donors. These results suggest that CAF-mediated secretion of PGE-2 is involved in regulating the functionality of NK cells by suppressing their activity, which promotes tumor growth (52).

#### Tumor-promoting inflammation

Inflammation is increasingly considered as a pivotal environmental factor contributing to tumor progression (13). Quantitative proteomic analysis has been performed on supernatants of CAFs isolated from an azoxymethane/dextran sodium sulfate mouse model of sporadic colon cancer (29). Protein networks indicated the association with desmoplastic and pro-inflammatory signatures. Pro-inflammatory signature is composed of several cytokines such as chemokine (C–C motif) ligand (CCL) 2 (MCP-1), CCL-8 (MCP-2), CCL-11 (Eotaxin-1), CCL-20, SAA-3, chemokine CXCL-2, CXCL-3, CXCL-5 (ENA-78), CXCL-12 (SDF-1), or CXCL-14. Furthermore, IL-6, IL-9, and osteopontin were up-regulated, whereas CSF1 was down-regulated in CAFs (14, 21, 29). CCL-2 is a chemokine involved in attracting macrophages into the tumor microenvironment and it also induces differentiation into an immunosuppressive M2 phenotype (17). CCL-8 attracts monocytes and lymphocytes. Addition of CCL-2 and CCL-8 to cultures of human CRC cells increased adhesion, migration, and invasion levels (29). CAF-derived chemokines CXCL-12 or CXCL-14 recruit bone marrow-derived cells, macrophages, and other immune cells into the tumor microenvironment, enabling tumor growth. Additionally, they recruit endothelial progenitor cells into tumors (21). IL-9 induced an increase in the proliferation of CRC cells (29), whereas IL-6 contributes to immune cell dysfunction and is known as a regulator of CD4^+^ T-cell differentiation (17). The expression of CCL-3 and its receptor CCR-5 were increased in the azoxymethane/dextran sodium sulfate mouse model. The numbers and size of colon tumors were lower in CCL-3- or CCR-5-deficient mice, in parallel with reduced CAF accumulation. *In vitro*, CCL-3 stimulated fibroblast proliferation and enhanced heparin-binding epidermal growth factor-like growth factor (HB-EGF) expression. These results indicate that CCL-3–CCR-5-mediated fibroblast accumulation may be required to induce colitis-associated carcinogenesis (53).

Cyclooxygenase (COX)-2, which is part of the pro-inflammatory signature of CAFs (23), is overexpressed in the majority of CRCs. COX-2-derived PGE-2 promotes tumor growth by inducing cancer cell proliferation, survival, migration/invasion, and by enhancing the development of a supportive tumor microenvironment. Additionally, PGE-2 suppresses effector T-cells (21). Normal intestinal fibroblasts activate signal transducer and activator of transcription 1 (STAT1) signaling in CRC cells and in contrast to CAFs, inhibit growth of cancer cells. CAFs lose the ability to trigger growth inhibitory STAT1 signaling in cancer cells, favoring tumor progression and suggesting STAT1 as a link between intestinal inflammation and CRC. PGE-2 has been shown to interfere with STAT1 activity, and may therefore contribute to the incapability of CAFs to activate STAT1 signaling in cancer cells (23). Pro-inflammatory cytokine LIF is secreted by cancer cells and fibroblasts treated with TGF-β1, which highlights the action of TGF-β1 as a driver of tumor-associated inflammation (54).

#### Activating invasion and metastasis

Incubation of CAFs with supernatants from CRC cells leads to hyperactivation of TGF-β signaling pathway. A strong autocrine regulatory loop augments the expression and secretion of TGF-β1 by CAFs (22). Stromal TGF-β signaling promotes metastasis formation (51). Two-thirds of mice bearing control KM12L4A colon tumors remained metastasis-free, whereas 10 of 11 mice inoculated with KM12L4A cells overexpressing TGF-β developed lung and/or liver metastasis. Because KM12L4a cells have an inactivated TGF-β pathway, enhanced metastasis initiation by TGF-β secretion must be due to changes in the tumor environment. Secretion of IL-11 by TGF-β stimulated CAFs triggers GP-130/STAT-3 signaling in cancer cells. This crosstalk suggests a survival advantage for metastatic cells. Metastasis initiation was blocked in mice treated with a pharmacological inhibitor of TGF-βR1. Metastases derived from TGF-β-secreting cell lines exhibited enhanced desmoplastic reaction with abundant phospho-SMAD2 accumulation in stromal cells and elevated expression of stromal TGF-β response signatures genes (51). TGF-β is involved in tumor metastasis development by increasing production of ECM and proteolytic enzymes (22). CAFs are the main source for connective tissue components of the ECM, such as collagens types I, III, IV, V, and XII, and proteoglycans (biglycan, fibromodulin, perlecan, and versican) (14, 29). Treatment of CAFs with TGF-β1 *in vitro* increased the expression of type I collagen, fibronectin, urokinase type plasminogen activator (u-PA), various MMPs (e.g., MMP-2 and MMP-9), and TIMPs in CAFs (22). Similarly, genes induced in CAFs, compared to normal resting colonic fibroblasts, include several tumor-promoting MMPs (MMP-2 and MMP-12) and ECM proteins implicated in invasion and metastasis, such as tenascin-C and laminin-B1 (14). The expression of MMPs in CAFs is generally high compared to CRC cells (22, 29). One of the main components of the basement membrane, type IV collagen, is a substrate of MMP-2 and MMP-9. The role of MMPs is apparent to be essential in invasion and metastasis (6, 22).

Snail1 expression was higher in colon CAFs than in normal fibroblasts. Snail1 is a transcriptional factor that plays an important role in epithelial–mesenchymal transition and in the achievement of invasive properties by epithelial cells. Co-culture of CAFs with CRC cells induced an increase in CRC cell migration and proliferation, which was correlated with mRNA SNAI1 expression levels in primary human CAFs (55). In another study, spheroids of CRC cells were co-cultured with CAFs in a collagen invasion experiment. Investigation of the F-actin organization revealed that spheroids in control conditions had smooth edges, whereas the spheroids in co-culture had an irregular perimeter with individual and collective cells invading the surrounding collagen matrix (24). Similarly, treatment of CRC cells with supernatants from CAFs induced a fivefold increase in a collagen invasion assay (28). In accordance, HCT116 cells treated with CAF supernatants showed a more elongated morphology compared to cells growing in supernatants from resting fibroblasts. CAFs increased the migration and invasion of CRC cells in Boyden chamber experiments and in a three-dimensional cell culture model (4, 16). FGFR-3 was identified as one of the receptor tyrosine kinases activated by CAF supernatants. CAFs hypersecreted FGF1 and increased FGF-1/FGFR-3 signaling led to migration and invasion (16). Herrera et al. demonstrated a clear association between the expression levels of α-SMA and promigratory stimulation of CRC cells. Most promigratory CAFs showed stem-cell markers. The association between typical stem-cell markers of CAFs and their promigratory effect on cancer cells was also demonstrated (4).

Elevated expression of PDGFRs on stromal CAFs is associated with advanced disease stage and an increased metastatic potential (21, 56). PDGF-stimulated fibroblasts increased migration and invasion of co-cultured CRC cells in a stanniocalcin-1 (STC-1)-dependent manner. STC-1 is a hypoxia-regulated and fibroblast-derived protein. In an orthotopic CRC model, there was a difference in the number of affected organs such that the tumors derived from the STC-1^−/−^ group showed a significantly lower number of affected organs per mouse. The foci were also significantly smaller than those in the control group and a significant decrease of lymphatic vessel density was observed in primary tumors with STC1^−/−^ mouse embryonic fibroblasts (56).

TGF-β1 stimulates the pro-invasive properties of human dermal fibroblasts by inducing LIF production. LIF-activated dermal fibroblasts gain cellular contractility and provide ECM remodeling via a crosstalk between the JAK1/STAT3 and RhoA/ROCK/MLC2 signaling pathways, reciprocally resulting in cancer cell invasion *in vitro* and *in vivo*. It was demonstrated that α-SMA expression, which depends on TGF-β signaling, is independent of LIF. Treatment with anti-TGF-β antibody had no effect on pro-invasive fibroblast activation, whereas LIF-blocking antibody completely abolished the pro-invasive effects of supernatants of squamous cell carcinoma. The latter activated STAT3 in fibroblasts only through LIF secretion. This indicates that monitoring α-SMA expression is not a criterion sufficient to reveal the presence of all the pro-invasive CAFs, which thus may lead to a biased prognosis for patients (54).

### Tumor-inhibiting characteristics of CAFs

Transgenic mice were generated with the ability to deplete α-SMA^+^ myofibroblasts in pancreatic cancer. Ptf1a^cre/+^; LSL-Kras^G12D/+^; Tgfbr2^flox/flox^ (PKT) mice, which develop spontaneous pancreas cancer, were crossed with α-SMA-tk transgenic mice to selectively target proliferating α-SMA^+^ myofibroblasts upon systemic ganciclovir administration. Depletion of CAFs led to invasive, undifferentiated tumors with enhanced hypoxia, EMT, and cancer stem cells, with reduced animal survival. Similarly, in pancreatic ductal adenocarcinoma patients, fewer CAFs also correlated with reduced survival. CAF depletion was associated with an overall decrease in the T_eff_/T_reg_ ratio associated with increased CTL-associated protein-4 expression. If CAFs are depleted, the tumor vasculature decreases, which may directly contribute to increased tumor hypoxia. Consequently, this promotes enhanced invasiveness and an undifferentiated phenotype of cancer cells. Thus, fibrosis associated with CAFs constitutes a protective response from the host rather than supporting an oncogenic role in pancreatic ductal adenocarcinoma patients (57). CAFs surrounding CRC cells demonstrated up-regulation of podoplanin, a mucin-type transmembrane glycoprotein, in CAFs *in vitro* and *in vivo*. Multivariate analysis of both disease-free survival and liver metastasis-free survival revealed that podoplanin expression is a significant indicator of good prognosis in patients with advanced CRC. Moreover, CRC cell invasion was enhanced by co-culture with CAFs that were treated with siRNA for podoplanin, suggesting a protective role against CRC cell invasion (58). However, reports about tumor-inhibiting characteristics of CAFs in CRC are scarce.

## Fibroblasts in Metastasis of CRC

We distinguish CAFs from the primary tumor and metastasis-associated fibroblasts (MAFs) in lymph node and distant metastases of CRC (mainly liver and lung). Here, the focus is put on liver metastases. MAFs may have other origins than CAFs and may exhibit other properties to prepare or maintain the (pre)metastatic niche.

**Figure 3:**
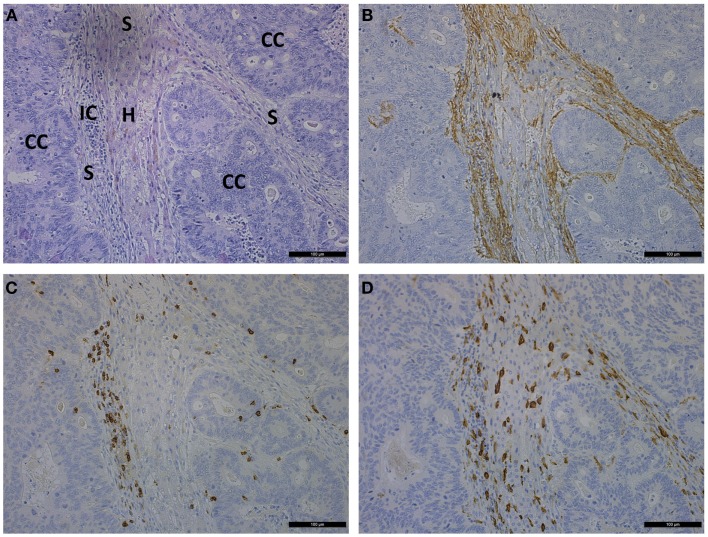
**Photomicrographs showing consecutive sections of liver metastasis of a CRC patient stained by hematoxylin and eosin (A) and stained immunohistochemically for α-SMA (B), CD8 (C), and CD163 (D)**. Scale bar is 100 μm. CC, cancer cells; IC, immune cells; S, stroma; H, hepatocytes.

Metastatic deposits of CRC within the liver are also characterized by a pronounced desmoplastic reaction associated with α-SMA^+^ MAFs (Figure 3) (59). HSCs are recognized as a major source of MAFs (59, 60). In mice experiments, HSCs were activated by various stimuli, e.g., PDGF-AB, HGF, and TGF-β and underwent transformation into MAFs when CRC cells migrated into the liver (59). Additional cell types such as resident liver fibroblasts/myofibroblasts or bone marrow cells are also known to generate MAFs (13, 60). Portal fibroblasts are present around the portal vein and fibroblastic cells around the central vein are called second-layered cells. The liver surface is covered with mesothelium, which is composed of mesothelial cells and fibroblastic cells called capsular fibroblasts. These fibroblastic cells around the veins and beneath the mesothelial cells have been suggested to differentiate into MAFs. Portal fibroblasts proliferate and differentiate into α-SMA-expressing MAFs and synthesize ECM proteins, similar to activated HSCs. Interestingly, unlike activated HSCs, portal fibroblasts do not respond to PDGF and their growth is rather inhibited by TGF-β (60).

Liver metastasis produces cancer-associated stroma organized into strands of α-SMA^+^ MAFs. Strands of MAFs are infiltrated by CD8 T-lymphocytes and CD163-activated macrophages. A discrete pattern of hepatocytes is massively surrounded by stroma and CRC metastatic cells (Figure 3). A comparative analysis between each primary tumor and a corresponding metastatic lesion showed that both displayed similar α-SMA values (18). However, in distant metastases of CRC those from the lung had fewer MAFs than those from the liver, peritoneum, or ovary. CRC cases with higher CAFs in center of the primary cancers had a tendency of higher MAFs in periphery (61). Desmoplastic reaction promotes growth and chemoresistance of CRC liver metastases (3).

Comparison of MAFs from liver metastasis of CRC with skin fibroblasts revealed up-regulation of genes of adhesion molecules, ECM/ECM remodeling molecules, proteases/protease inhibitors, and several growth factors and survival cytokines, including PDGFA, FGF-1, IGFBP-7, IGFBP-5, CTGF, prostate differentiation factor, VEGF, TGF-β2, monocyte chemotactic protein-1 (MCP-1), IL-6, osteoprotegerin, follistatin, follistatin-related gene, COX-2, and TGF-β (20). Supernatants from MAFs from liver metastasis of CRC enhanced proliferation of HCT116 CRC cells, more than supernatant from uninvolved skin fibroblasts. However, in comparison to liver fibroblasts, the results were not significant, and no data are available where MAFs are compared with CAFs (20). The colorectal metastases display immunoreactivity for TNF-α in cancer cells, whereas stromal fibroblasts are negative. Increased migration of CRC cells induced by TNF-α-treated MAFs from human liver metastases has been demonstrated. These TNF-α-treated MAFs showed an increased expression of IL-6, MCP-1, and ICAM-1 (62). In liver metastasis, periostin immunoreactivity was observed in the capsular stroma. Whereas there was little periostin immunoreactivity in normal liver tissue, including the portal areas. As described previously, periostin promotes growth and colony size of CRC cells (12).

In liver metastases from CRC, α-SMA^+^ MAFs derived from HSCs, surrounding the liver metastasis foci, secrete SDF-1. Immunohistochemistry was performed to examine the expression of CXCR4, the receptor of SDF-1 ligand, in human samples of CRC and liver metastasis. It was shown that the proportion of CXCR4 expression in the primary tumors with liver metastasis was higher than in those without liver metastasis. Moreover, there are more CXCR4^+^ cells at the metastatic site in the liver compared with the primary sites. Stimulation with SDF-1 increased the number of invading HCT116 cells *in vitro*. Additionally, HCT116 cells became resistant to apoptosis by SDF-1 stimulation. This phenomenon suggests that liver metastases could be formed with the help of MAFs secreting SDF-1. CRC cells may drift to the liver, where MAFs secrete SDF-1 and promote tumor formation (59). SDF-1 and CXCR4 expression were also significantly associated with lymph node metastasis, tumor stage, and survival of CRC patients. Most of the CRCs with lymph node metastases showed strong SDF-1α expression, not only in the primary lesion but also in their lymph node metastases (63).

During metastatic colonization, cancer cells may instruct the stroma of the host organ by either secreting TGF-β or recruiting TGF-β-producing cells such as macrophages, CAFs, or platelets. IL-11 was among the genes highly up-regulated by TGF-β in colon fibroblast cultures and in experimental metastasis generated from TGF-β-secreting KM12L4a cells. CAFs were the only source of IL-11 in tumors. IL-11 enhances metastasis initiation and in this way the liver metastatic potential of CRC cells. This involves STAT3 signaling in CRC cells, which suppresses apoptotic stimuli encountered during the colonization of the metastatic site. Nuclear phospho-STAT3 accumulation in primary CRC samples associates to advanced disease and poor outcome (51).

## Conclusion and Future Perspectives

The importance of CAFs in cancer progression through multiple interactions with cancer cells and other cells in the tumor environment suggests the use of CAF markers as prognostic biomarkers. CAFs and their products should be considered as novel therapeutic targets in primary and metastatic disease. The abundance of α-SMA-expressing CAFs in CRC is suggested as a useful indicator of poor prognosis. However, these results were restricted to stage II and III CRCs (18). In patients with advanced metastatic disease, increased stromal FAP is an adverse prognostic marker (64). A significant correlation was observed between stromal FAP-α and SDF-1 mRNA levels, primarily expressed by CAFs, after pre-operative chemoradiotherapy in rectal cancer patients (65). Positive gene expressions of FAP-α and SDF-1 were significantly correlated with distant recurrence and poor probability of recurrence-free and overall survival. Patients with elevated serum SDF-1 levels had equally poor overall survival as those with positive stromal SDF-1 gene expression (65). In CRC patients, the combination of calumenin with cadherin 11 expressed by CAFs displayed a significant association with disease-free survival and overall survival (29). In conclusion, detection and monitoring of CAF reactions may better define prognosis. Future research will determine if CAFs are potential target candidates in cancer management protocols.

## Conflict of Interest Statement

The authors declare that the research was conducted in the absence of any commercial or financial relationships that could be construed as a potential conflict of interest.
